# Point-of-care Ultrasound Diagnosis of Cardiac Myxoma

**DOI:** 10.5811/cpcem.47142

**Published:** 2025-07-24

**Authors:** Joseph Brutico, Daniel Kreider

**Affiliations:** Wellspan York Hospital, Department of Emergency Medicine, York, Pennsylvania

**Keywords:** POCUS, cardiac myxoma, echocardiography, case report

## Abstract

**Introduction:**

Cardiac myxomas are rare benign tumors of the heart that can become clinically relevant due to cardiovascular effects. Diagnosis can be challenging due to non-specific presenting symptoms. Point-of-care ultrasound (POCUS) provides a convenient first-line screening modality.

**Case Presentation:**

A 65-year-old male with a history of tobacco use presented to the emergency department (ED) with a month of progressive dyspnea with exertion and hematemesis. Cardiac POCUS and pulmonary computed tomography with angiography revealed a left atrial mass consistent with a cardiac atrial myxoma. The patient underwent coronary artery bypass grafting with excision of the left atrial myxoma via right atriotomy and atrial septal defect repair.

**Discussion:**

Presented is a case of a patient presenting with progressive dyspnea diagnosed with a cardiac myxoma using POCUS in the ED. Cardiac myxomas have a wide variety of clinical presentations, and emergency physicians must maintain a high index of suspicion. Point-of-care-ultrasound is well suited for early diagnosis of this unique pathology. Surgical resection and tumor histopathology remain the mainstay of treatment.

## CASE PRESENTATION

**Image 1:**
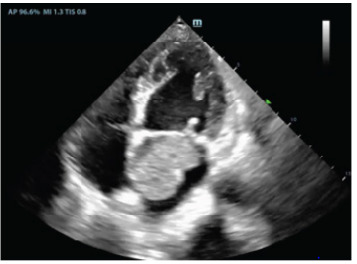
Computed tomography coronal view demonstrating a 3 x 3 x 5 centimeter left atrial mass (arrow) later confirmed to represent an atrial myxoma.

**Image 2:**
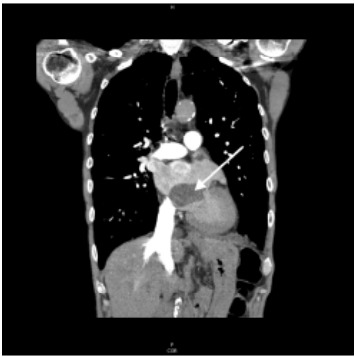
Computed tomography axial view demonstrating a 3 x 3 x 5 centimeter left atrial mass (arrow) later confirmed to represent an atrial myxoma.

A 65-year-old male with a history of tobacco use presented to the ED with a month of progressive dyspnea with exertion and hematemesis. On review of systems, the patient reported unintentional weight loss and night sweats. Examination revealed a cachectic male appearing older than stated age with slight tachypnea. Cardiac point-of-care ultrasound (POCUS) (Video 1) and pulmonary computed tomography (CT) with angiography (Images 1 and 2) revealed a 3 x 3 x 5 centimeter (cm) left atrial mass consistent with a cardiac atrial myxoma, which was later confirmed by pathology.

After left heart catheterization, the patient underwent coronary artery bypass grafting with excision of the left atrial myxoma via right atriotomy and atrial septal defect repair.

## DISCUSSION

Cardiac myxomas are the most common primary cardiac tumor. A vast majority of myxomas (80–90%) arise from the left atrium with fewer involving the right atrium (7–20%). Rarely, myxomas may be biatrial or arise from the ventricles.^1^ Symptoms vary and typically arise due to obstruction (heart failure), invasion of myocardial tissue (arrhythmias), or embolization (ischemia).^2^ Embolization occurs in up to 40% and is associated with villous tumors, size less than 4.5 cm, and valvular site of origin.^3^ Management begins with confirmation of diagnosis as common mimics include mural thrombi and valvular vegetations.^2^ While echocardiography is the diagnostic modality of choice, CT or cardiac magnetic resonance imaging may also be considered. Surgical resection and tumor histopathology remain the mainstay of treatment, with postoperative 30-day mortality < 5%.^4^ The most common complication is cardiac arrhythmia with reported rates of up to 20%.^5^ This clinical pathology requires a high index of suspicion and use of multiple diagnostic modalities including cardiac POCUS.

*CPC-EM Capsule*

What do we already know about this clinical entity?

*Cardiac myxomas are rare benign tumors of the heart that can present with a wide range of clinical symptoms. Management includes confirmation of diagnosis and surgical resection*.

What is the major impact of the image(s)?

*This case highlights cardiac myxoma as an important diagnosis to consider in the evaluation of a patient with dyspnea and has unique and recognizable echocardiographic findings*.

How might this improve emergency medicine practice?

*With a high level of suspicion, emergency physicians can accurately recognize this disease state using point-of-care ultrasound, leading to appropriate management and good long-term survival*.

## Figures and Tables

**Video:** Cardiac point-of-care ultrasound apical four-chamber view demonstrating a left atrial mass later confirmed to represent an atrial myxoma.
